# Cardiorespiratory Fitness in Childhood and Adolescence Affects Future Cardiovascular Risk Factors: A Systematic Review of Longitudinal Studies

**DOI:** 10.1007/s40279-018-0974-5

**Published:** 2018-08-24

**Authors:** Stijn Mintjens, Malou D. Menting, Joost G. Daams, Mireille N. M. van Poppel, Tessa J. Roseboom, Reinoud J. B. J. Gemke

**Affiliations:** 1 Department of Pediatrics, Emma Children’s Hospital, Amsterdam Reproduction and Development, Amsterdam Public Health Research Institute, Amsterdam UMC, Vrije Universiteit Amsterdam, De Boelelaan 1118, Room KTC 4-021, 1081 HZ Amsterdam, The Netherlands; 2 Department of Gynecology and Obstetrics, Department of Clinical Epidemiology, Biostatistics and Bioinformatics, Amsterdam Reproduction and Development, Amsterdam Public Health Research Institute, Amsterdam UMC, University of Amsterdam, Meibergdreef 5, 1105 AZ Amsterdam, The Netherlands; 3 Medical Library AMC, Amsterdam UMC, University of Amsterdam, Meibergdreef 5, 1105 AZ Amsterdam, The Netherlands; 4 Amsterdam Public Health Research Institute, Amsterdam UMC, Vrije Universiteit Amsterdam, Van der Boechorststraat 7, 1081 BT Amsterdam, The Netherlands; 5 Institute of Sport Science, University of Graz, Mozartgasse 14, 8010 Graz, Austria

## Abstract

**Background:**

Although cardiorespiratory fitness (CRF) in childhood and adolescence may be linked to future cardiovascular health, there is currently limited evidence for a longitudinal association.

**Objectives:**

To provide a systematic review on the prospective association between CRF in childhood and adolescence and cardiovascular disease (CVD) risk factors at least 2 years later.

**Methods:**

Using a systematic search of Medline, Embase, and SPORTDiscus, relevant articles were identified by the following criteria: generally healthy children and adolescents between 3 and 18 years of age with CRF assessed at baseline, and a follow-up period of ≥ 2 years. The outcome measures were CVD risk factors. We appraised quality of the included articles with STROBE and QUIPS checklists.

**Results:**

After screening 7524 titles and abstracts, we included 38 articles, assessing 44,169 children and adolescents followed up for a median of 6 years. Eleven articles were of high quality. There was considerable heterogeneity in methodology, measurement of CRF, and outcomes, which hampered meta-analysis. In approximately half of the included articles higher CRF in childhood and adolescence was associated with lower body mass index (BMI), waist circumference, body fatness and lower prevalence of metabolic syndrome in later life. No associations between CRF in childhood and adolescence and future waist-to-hip ratio, blood pressure, lipid profile, and glucose homeostasis were observed.

**Conclusion:**

Although about half of the included articles reported inverse associations between CRF in childhood and adolescence and future BMI, body fatness, and metabolic syndrome, evidence for other CVD risk factors was unconvincing. Many articles did not account for important confounding factors such as adiposity. Recommendations for future research include standardizing the measurement of CRF, i.e. by reporting *V*O_2max_, using standardized outcome assessments, and performing individual patient data meta-analyses.

**Electronic supplementary material:**

The online version of this article (10.1007/s40279-018-0974-5) contains supplementary material, which is available to authorized users.

## Key Points

**Table:** 

A high level of physical fitness in childhood and adolescence is associated with lower risks of future overweight, fatness, and metabolic syndrome.
There is no convincing evidence linking a high level of physical fitness in childhood and adolescence to healthier future blood pressure, lipid profile, or glucose homeostasis.

## Introduction

Cardiovascular disease (CVD) remains the leading cause of death in adults worldwide [1]. While CVD often becomes clinically apparent during late adulthood, there is mounting evidence that the disease originates in early life [2–6]. For example, higher blood pressure in childhood predicts poorer cardiovascular health in mid-adulthood, and high body mass index (BMI) in early age reduces later cardiovascular health even further [7]. A high level of physical fitness is associated with reduction of CVD risk factors in adults [8, 9], and the American Heart Association recognizes low levels of fitness as an important risk factor for CVD [10]. However, there is limited evidence of the relation between physical fitness at a young age and CVD risk factors later in life. Potentially, physical fitness in childhood and adolescence is a useful early predictor of CVD risk factors and overall health [11].

Physical fitness comprises various entities, such as muscular strength, agility, balance, and cardiorespiratory fitness (CRF); of these CRF is most strongly associated with health outcomes [12]. The gold standard to test CRF is by exercising until voluntary exhaustion with direct measurement of the maximum volume of oxygen consumption (*V*O_2max_), and requires specialized equipment. Fortunately, many field-tests, sub-maximal tests, and even predicting equations provide reliable estimates of CRF in a wide range of settings and participants [10, 13, 14], thus making them implementable in many areas. By assessing CRF, a quantification of individual capacities of numerous body systems is provided [15, 16], and thus CRF provides a quantification of total body health.

Cross-sectional studies in children and adolescents show strong correlations of CRF with CVD risk factors [17–19]; however, it has been suggested that these associations could be more readily explained by a child’s adiposity [20]. It is likely that children and adolescents who are active have better CRF, but being active also directly affects adiposity [21]. Some studies have linked higher levels of physical activity (PA) to better CRF, but correlations were moderate [22, 23]. This could be explained by the fact that a large proportion of the variability in CRF is genetically determined, as seen in the heterogeneity in the response to regular exercise in individuals [24, 25]. Hence, the genetic component of CRF may affect the ability of the body to resist the effects of an unhealthy lifestyle, or to be more susceptible to the beneficial effects of regular PA, protecting against future CVD development.

Indeed, the potential health benefits of high levels of CRF in early life for cardiovascular function in later life have been demonstrated in a large number of publications. Thus far, due to the difficulties in following children and adolescents without potential risk factors into late adulthood when CVD becomes apparent, follow-up has focused on CVD risk factors. These include obesity, high blood pressure, high levels of cholesterol and triglycerides, and insulin resistance as proxies for CVD [26, 27]. Previous reviews have shown that reduced CRF is associated with higher prevalence of CVD risk factors; however, these reviews have some important limitations [12, 28, 29]. First, most evidence is based on cross-sectional studies, which makes it impossible to assess directionality. Prospective studies are more suitable to provide insight into the direction of the association, but thus far no conclusive prospective systematic review has been published. Second, some of these reviews included articles with only adults at baseline. This might interfere with the validity of the association between early life fitness and later CVD risk factors, as these risks are more prevalent in the adult population. From a preventive viewpoint, the relation between CRF in childhood and adolescence and the development of CVD risk factors is paramount. Therefore, we aimed to systematically review the current evidence for a prospective association between CRF in childhood and adolescence and CVD risk factors.

## Methods

This review has been registered in PROSPERO (CRD42015025064). The methodology applied in this review adhered to the guidelines outlined in the Preferred Reporting Items for Systematic Reviews and Meta-Analysis (PRISMA) statement [30].

### Literature Search

A comprehensive systematic literature search was constructed with the help of a clinical librarian and performed in the databases Medline, Embase, and SPORTDiscus from inception until 23 October 2017. Key concepts derived from a scoping search using among others forward/backward citation tracking in Google Scholar and searching the WHO-ICTRP search portal, are embodied as follows in the systematic search strategy: (“Children aged 3 till 18 years” AND (“fitness” OR “cardiorespiratory tests” OR “cardiorespiratory test parameters”) AND “prospective studies”) OR “young hearts study”.

Key papers identified in the scoping search were all retrieved by the systematic search strategy. Furthermore, conference abstracts and books were filtered out when possible in the search. No other filters were used. The search syntax was adapted to the indexing terms of each database. The full search strategy is included as Electronic Supplementary Material Appendix S1.

### Eligibility Criteria

Articles were included if they met the following criteria: (1) Longitudinal prospective study design with a follow-up period of ≥ 2 years; (2) participants at baseline were aged 3 up to and including 18 years (3) the study population was based on a general healthy population, irrespective of BMI; (4) physical fitness was assessed at baseline; and (5) it was an original article published in a peer-reviewed journal. There were no restrictions on type of fitness testing, i.e., objectively measured CRF, field tests, and composite tests were considered, since the aim of this review was to provide an overall estimate of the association between CRF and future CVD risk factors. There was no restriction on language; if after screening of the (English) title and abstract the article was deemed eligible, it was then translated. The outcomes measures included were confined to the following three categories: (1) anthropometry (e.g., BMI, overweight/obesity status, waist and/or hip circumference, skinfolds, percentage body fat (%BF), fat-free mass (FFM)), (2) circulatory system (e.g., systolic/diastolic blood pressure (SBP/DBP), arterial stiffness, pulse wave velocity (PWV), intima media thickness (IMT), cardiovascular events), and (3) metabolic (e.g., lipid profiles, glucose levels, insulin sensitivity, low grade infection). Also, articles reporting on the prevalence of metabolic syndrome or CVD risk scores were included.

### Study Selection Process

First, two reviewers (SM and MM) independently screened titles and abstracts of the articles retrieved by the search strategy for eligibility. Second, full texts of articles were acquired. The same two reviewers independently screened the full texts of articles to determine whether to include the article based on the inclusion criteria. During both stages discrepancies were discussed, and when no consensus was reached a third reviewer (RG) made the final decision about inclusion.

### Data Extraction and Assessment

Two reviewers (SM and MM) independently extracted data from the included articles using a pilot-tested standardized form. The following information was extracted: (1) study aim; (2) study design; (3) characteristics of cohort/participants at baseline; (4) characteristics of subjects with complete follow-up; (5) type of fitness test and representation of result; (6) the primary outcome of the study; (7) outcome of interest for this review and what confounders were corrected for. When data were unclear or not reported, attempts were made to contact authors. Inconsistencies in the extracted data were discussed between reviewers until consensus was reached.

Quality assessment was done independently by the same two reviewers. Based upon the STROBE quality assessment tool [31], the reporting in each article was scored as good, sufficient, or poor. Bias was assessed with the Quality In Prognostic Studies tool (QUIPS) [32]. This tool assesses the following six areas: participation, attrition, prognostic factor measurement, confounding measurement and account, outcome measurement, and analysis and reporting. Each of the potential bias domains was rated as having high, moderate, or low risk of bias. Based on both the QUIPS and STROBE score, studies were rated as indicated in Table 1. The lowest score in QUIPS or STROBE determined the overall rating. Discrepancies in quality and risk of bias assessment were discussed between reviewers until consensus was reached.

**Table 1:** Quality assessment classification based on QUIPS and STROBE tools

Rating	QUIPS	STROBE
High quality	All items scored as low risk, or at most one item scored as moderate risk	Good
Moderate quality	Two items scored as moderate risk and other items scored as low risk, or five items scored as low risk and one item scored as high risk	Sufficient
Low quality	Three or more items scored as moderate risk or at least one item scored as moderate and one or more items scored as high risk	Poor

*QUIPS* Quality In Prognostic Studies, *STROBE* Strengthening the Reporting of Observational Studies in Epidemiology

### Data Synthesis

**Fig. 1:**
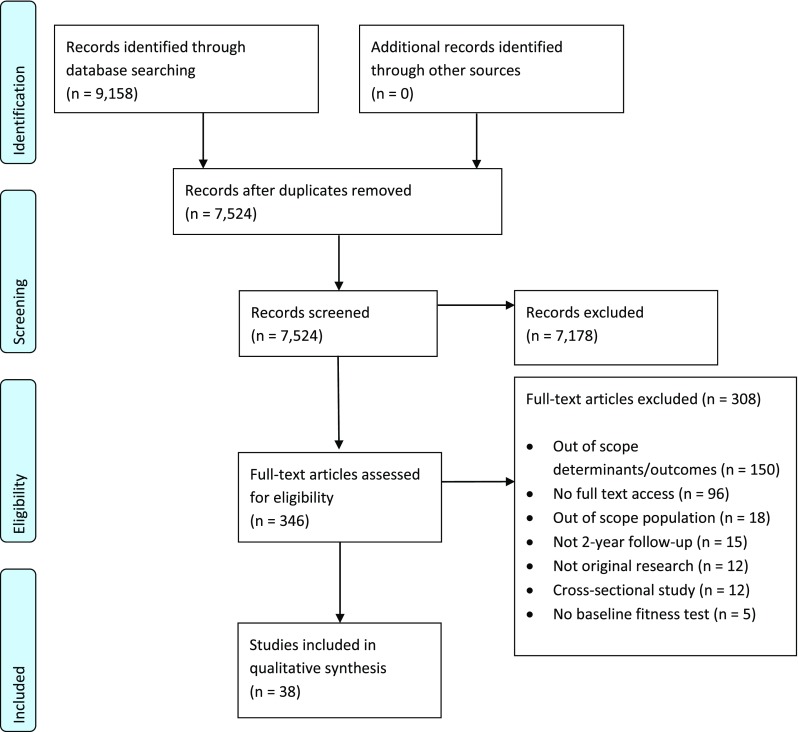
PRISMA flow diagram

A flowchart of the included articles is presented according to the PRISMA guidelines [30] in Fig. 1. Relevant characteristics of the articles are presented in Table 2. Table 3 presents the outcomes of the risk of bias and quality assessment. Although our aim was to perform a meta-analysis and present pooled data, the heterogeneity of the included articles precluded execution of this plan. Hence, we present a narrative data-synthesis. In Table 4 a summary of the reported associations is presented per outcome and stratified per sex where possible.

**Table 2:** Summary of characteristics and findings of included articles

Reference	Population baseline (*N*, % male, age, country)	Follow-up duration and *N* at follow-up	Type and method of fitness testing	Outcomes of interest	Adjustment for confounders	Relevant result
Aires et al. (2010) [63] Portuguese cohort	*N* = 345, 50% boys, age 11–19 years, Portugal	3 years, *N* = 225	Composite Z score of curl-ups, push-ups and 20 m shuttle run; ‘Low-fit’ below first tertile vs. ‘High-fit’ ≥ first tertile	BMI and ΔBMI	N/A	**BMI** Low-fit 23.3 kg/m^2^ vs. high-fit 21.5 kg/m^2^, (*p* = unknown) **ΔBMI** High-fit from 2006 to 2008 = 1.28 ± 3.48 Low-fit from 2006 to 2008 = 1.44 ± 1.76 (*p* = unknown)
Aires et al. (2010) [64] Portuguese cohort	*N* = 345, 50% boys, age 14 ± 1.38 years (range 11–16 years), Portugal	2 years, *N* = not specified	Score of curl-ups, push-ups and 20 m shuttle run; Characterized as healthy zone or under healthy zone	BMIc	Model 1: Adjusted for time Model 2: Also, mother’s education, curl-up, push-up, back-saver sit&reach, screen time, school commute, physical activity index	**BMIc** 1) *B* 0.435 (95% CI 0.116; 0.754) *P* = 0.008 2) *B* 0.766 (95% CI 0.289; 1.244) *P* = 0.002
Boreham et al. (2002) [56] Northern Ireland Young Hearts Project	*N* = 1015, 50% boys, age group 1: 12.5 ± 0.3, group 2: 15.5 ± 0.3 years, Northern Ireland	10 years, *N* = 459	20 m-MSRT, number of complete laps	SumSF SBP, DBP TC, HDL-C, TC:HDL	Social class and sexual maturity	**SumSF** (M) 12 years *β* − 0.37 (*P* < 0.01); 15 years *β* − 0.38 (*P* < 0.01) (F) 12 years *β* − 0.41 (*P* < 0.01); 15 years *β* − 0.34 (*P* < 0.01)
						**SBP** (M) 12 years β 0.05 NS, 15 years β 0.02 NS, (F) 12 years *β* − 0.018 NS, 15 years *β* − 0.07 NS **DBP** (M) 12 years *β* − 0.06 NS, 15 years β 0.08 NS, (F) 12 years *β* − 0.01 NS, 15 years *β* − 0.10 NS
						**TC** (M) 12 years *β* − 0.12 NS, 15 years β 0.00 NS (F) 12 years *β* − 0.02 NS 15 years β 0.00 NS **HDL-C** (M) 12 years β 0.14 NS, 15 years β 0.21 NS, (F) 12 years *β* − 0.02 NS, 15 years β 0.00 NS **TC:HDL** (M) 12 years *β* − 0.15 NS, 15 years *β* − 0.22 (*P* = 0.05), (F) 12 years *β* − 0.27 (*P* = 0.01), 15 years *β* − 0.06 NS
Twisk et al. (1999) [54] Northern Ireland Young Hearts Project	*N* = 509, 49% boys, age 12 years, Northern Ireland	3 years, *N* = 459	20 m-MSRT, number of completed laps (divided into a ‘risk’ quartile versus the other three ‘non-risk’ quartiles)	SumSF	Maturity and SES	**SumSF** (M) OR 5.46 (95% CI 3.42; 8.73) (F) OR 4.00 (95% CI 2.46; 6.51)
				DBP		**DBP** (M) OR 1.62 (95% CI 1.04; 2.53) (F) OR 1.05 (95% CI 0.71; 1.56) NS
				TC:HDL		**TC:HDL** (M) OR 1.66 (95% CI 1.12; 2.45) (F) OR 1.76 (95% CI 1.11; 2.81)
Ferreira et al. (2005) [57] Amsterdam Growth and Health Longitudinal study	*N* = 450, 48% boys, age 13.1 ± 0.8 years, the Netherlands	24 years, *N* = 364	Test not specified, used *V*O_2max_ in ml/min/kg	MetS (≥ 3 out of 5 risk factors) (1) SBP ≥ 130 mmHg and/or DBP ≥ 85 mmHg; (2) HDL cholesterol (M: < 40 mg/dl, F: < 50 mg/dl; (3) TG > 150 mg/dl; (4) HbA1c > 6.1% (5) WC M: > 94 cm, F: > 80 cm	Sex	No differences at adolescents in CRF in those with and without MetS at 36 years (extracted from graph)
Ferreira et al. (2002) [58] Amsterdam Growth and Health Longitudinal study	*N* = 450, 49% boys, age 13.1 ± 0.8 years (range 13–16 years), the Netherlands	24 years, *N* = 154	Maximal running test on treadmill to measure *V*O_2max_ in ml/min/kg	Ultrasound carotid arterial properties: intima media thickness; diameter; distension; distensibility; compliance coefficient; Young’s elastic modulus	Model 1. Sex Model 2. Also body height, body weight, sum of four skinfolds, mean blood pressure (systolic and diastolic blood pressure instead, in analyses with IMT), total and HDL-C, resting heart rate and smoking status	**Carotid arterial properties:** IMT 1) (M) β −0.244 (*P* = 0.035) (F) β 0.170 (*P* = 0.135) NS; 2) (M) β −0.381 (*P* = 0.012) (F) β 0.204 (*P* = 0.138) NS Diameter 1) (M) β −0.058 (*P* = 0.622) NS (F) β 0.145 (*P* = 0.202) NS; 2) (M) β −0.189 (*P* = 0.201) NS (F) β 0.149 (*P* = 0.242) NS Distension 1) (M) β −0.094 (*P* = 0.422) NS (F) β 0.150 (*P* = 0.188) NS; 2) (M) β −0.087 (*P* = 0.557) NS (F) β 0.105 (*P* = 0.414) NS Distensibility coefficient 1) (M) β −0.024 (*P* = 0.839) NS (F) β 0.163 (*P* = 0.150) NS; 2) (M) β 0.024 (*P* = 0.877) NS (F) β 0.107 (*P* = 0.406) NS Compliance coefficient 1) (M) β −0.038 (*P* = 0.743) NS (F) β 0.192 (*P* = 0.089) NS; 2) (M) β −0.077 (*P* = 0.606) NS (F) β 0.157 (*P* = 0.213) NS Elastic modulus 1) (M) β 0.124 (*P* = 0.288) NS (F) β −0.217 (*P* = 0.054) NS; 2) (M) β 0.112 (*P* = 0.468) NS (F) β −0.189 (*P* = 0.154) NS
				Femoral arterial properties: diameter; distension; distensibility; compliance coefficient		
						**Femoral arterial properties:** Diameter 1) β 0.382 (< 0.001); 2) β 0.252 (*P* = 0.026) Distension 1) β −0.188 (*P* = 0.171) NS; 2) *β* − 0.395 (*P* = 0.016) Distensibility coefficient 1): β −0.214 (*P* = 0.088) NS; 2) *β* − 0.344 (*P* = 0.024) Compliance coefficient 1) β −0.029 (*P* = 0.832) NS; 2) β −0.257 (*P* = 0.121) NS
Twisk et al. (2002) [61] Amsterdam Growth and Health Longitudinal study	*N* = 307, 48% boys, age 13.1 ± 0.8 years, the Netherlands	20 years, *N* = 277	Maximal treadmill test to measure *V*O_2max_, expressed as absolute (L/min), ml/min/kg and maximal slope (%) of the treadmill (at 8 km/h) A) fitness at age 13 and B) “Maintained” exposure: the average *V*O_2max_ over the first four annual measurements (between 13 and 16 years)	WHR, WC	Sex and age, if interaction with sex than separate for sex	**WHR** *V*O_2max_ (absolute), A) β − 0.03 NS; B) *β* − 0.05 NS; *V*O_2max_ (per kg), A) *β* − 0.01 NS; B) β 0.08 NS Maximal slope, A) β 0.00 NS; B) β 0.06 NS **WC** *V*O_2max_ (absolute), A) β 0.11 NS; B) (M) *β* − 0.21 NS and (F) *β* − 0.34 *P* < 0.01 *V*O_2max_ (per kg), A) (M) *β* − 0.01 NS and (F) *β* − 0.26 *P* < 0.01. B) *β* − 0.14 NS Maximal slope, A) (M) β 0.06 NS and (F) *β* − 0.23 *P* < 0.01; B) *β* − 0.10 NS
				SumSF		
				SBP, DBP		
				TC, HDL-C, and TC:HDL		
						**SumSF** *V*O_2max_ (absolute), A) *β* − 0.02 NS; B) β 0.09 NS *V*O_2max_ (per kg), A) *β* − 0.29 *P* < 0.01; B) *β* − 0.34 *P* < 0.01 Maximal slope, A) (M) *β* − 0.011 NS and (F) *β* − 0.25 *P* < 0.01; B) *β* − 0.32 *P* < 0.01
						**SBP** *V*O_2max_ (absolute), A) (M) *β* − 0.27 *P* < 0.05 and (F) β 0.1 NS; B) (M) *β* − 0.16 NS and (F) β 0.19 NS *V*O_2max_ (per kg), A) *β* − 0.01 NS; B) *β* − 0.25 *P* < 0.05 Maximal slope, A) *β* − 0.04 NS; B) β 0.06 NS **DBP** *V*O_2max_ (absolute), A) *β* − 0.03 NS; B) (M) *β* − 0.06 NS and (F) β 0.16 NS. *V*O_2max_ (per kg), A) *β* − 0.09 NS; B) *β* − 0.11 NS Maximal slope, A) *β* − 0.12 NS; B) β 0.05 NS
						**TC** *V*O_2max_ (absolute), A) β 0.04 NS; B) β 0.05 NS. *V*O_2max_ (per kg), A) (M) *β* − 0.17 NS and (F) β 0.11 NS; B) − 0.27 NS Maximal slope A) (M) *β* − 0.19 *P* < 0.05 and (F) β 0.05 NS; B) *β* − 0.23 *P* < 0.05 **HDL-C** *V*O_2max_ (absolute), A) β 0.01 NS; B) β 0.01 NS. *V*O_2max_ (per kg), A) β 0.04 NS; B) β 0.13 NS Maximal slope A) *β* − 0.05 NS; B) β 0.05 NS **TC:HDL ratio** *V*O_2max_ (absolute), A) β 0.03 NS; B) β 0.03 NS. *V*O_2max_ (per kg), A) *β* − 0.07 NS; B) *β* − 0.26 *P* < 0.05 Maximal slope A) (M) *β* − 0.11 NS and (F) β 0.09 NS; B) *β* − 0.17 NS
Grontved et al. (2011) [42] European Youth Heart Study	*N* = 589, 45% boys, age boys 9.8 ± 0.4, girls 9.7 ± 0.4 years, Denmark	6 years, *N* = 226	Graded maximal aerobic fitness test: A) Stage 2 exercise SBP, B) Last completed stage SBP, C) Slope of intensity-SBP function, D) HR at stage 2, E) HR at last completed stage, F) Rate pressure product (RPP) stage 2, G) RPP last completed stage	Resting SBP	Model 1: Age and sex Model 2: also childhood levels of resting SBP and DBP Model 3: also BMI, CRF, TC, HDL-C, TG, insulin and glucose at baseline	**SBP** *β* 1) A) *B* = 0.19 (95% CI 0.11; 0.27); B) *B* = 0.11 (95% CI 0.04; 0.18); C) *B* = 5.75 (95% CI − 0.17; 11.68) NS; D) *B* = 0.05 (95% CI − 0.01; 0.12) NS; E) *B* = 0.07 (95% CI − 0.02; 0.16) NS; F) *B* = 0,07 (95% CI 0.04; 0.11); G) *B* = 0.05 (95% CI 0.02; 0.08) 2) A) *B* = 0.09 (95% CI 0.002; 0.18); B) *B* = 0.04 (95% CI − 0.04; 0.11) NS; C) *B* = 4.46 (95% CI − 1.08; 10.01) NS; D) *B* = 0.05 (95% CI − 0.01; 0.11) NS; E) *B* = 0.07 (95% CI − 0.02; 0.16) NS; F) *B* = 0,05 (95% CI 0.01; 0.08); G) *B* = 0.02 (− 0.01; 0.05) NS 3) A) *B* = 0.09 (95% CI − 0.003; 0.18) NS; B) *B* = 0.03 (95% CI − 0.04; 0.11) NS; C) *B* = 5.52 (95% CI − 0.09; 11.13) NS; D) *B* = 0.06 (95% CI − 0.01; 0.13) NS; E) *B* = 0.06 (95% CI − 0.03; 0.16) NS; F) *B* = 0,05 (95% CI 0.01; 0.09); G) *B* = 0.02 (− 0.01; 0.05) NS
Grontved et al. (2013) [59] European Youth Heart Study	First cohort *N* = 429, Second cohort *N* = 444, approx. 47% boys, age 15 years, Denmark	6 or 12 years, *N* = 317	Maximal progressive ergometer bicycle test. Estimated *V*O_2max_ in ml/min/kg	Fasting glucose, insulin (%change), HOMA-IR (%change), and HOMA-B (%change)	Model 1: Adolescent age, adulthood age, sex, recruitment period Model 2: Also parental educational level, current smoking, family history of diabetes, soft drinks intake, and fruit and vegetables intake. Model 3: Also muscular strength Model 4: Also WC	**Glucose** 1) *B* = − 0.04 (95% CI − 0.09; 0.01) NS 2) *B* = − 0.03 (95% CI − 0.08; 0.03) NS 3) *B* = − 0.02 (95% CI − 0.08; 0.04) NS 4) *B* = − 0.02 (95% CI − 0.09; 0.05) NS **Insulin** 1) *B* = − 17.0 (95% CI − 22.7; − 10.9) 2) *B* = − 16.6 (95% CI − 22.5; − 10.2) 3) *B* = − 12.8 (95% CI − 19.2; − 5.8) 4) *B* = 11.4 (95% CI − 19.0; − 3.2) **HOMA-IR** 1) *B* = − 17.8 (95% CI − 23.9; − 11.3) 2) *B* = − 17.3 (95% CI − 23.6; − 10.5) 3) *B* = − 13.3 (95% CI − 20.1; − 5.9) 4) *B* = − 12.1 (95% CI − 20.1; − 3.2) **HOMA-B** 1) *B* = − 13.2 (95% CI − 18.7; − 7.4) 2) *B* = − 13.2 (95% CI − 18.8; − 7.2) 3) *B* = − 10.0 (95% CI − 16.1; − 3.4) 4) *B* = − 9.2 (95% CI − 16.5; − 1.4)
Andersen et al. (2004) [38]	*N* = 305, 44% boys, age 16–19 years, Denmark	8 years, *N* = 235	Maximal progressive cycle test to measure *V*O_2max_ in ml/min/kg, divided into quartiles	Metabolic syndrome: ≥ 2 risk factors (upper quartile of TC:HDL, TG, SBP and body fat)	Fitness at follow-up	**MetS** No significant relationships between fitness level at baseline and being a case (clustered risk CVD) at follow-up both with and without adjustment for fitness level at second examination (no data shown)
Andersen et al. (2011) [39]	*N* = 706, %boys N/A, age boys 6.8 ± 0.4, girls 6.7 ± 0.3 years, Denmark	2.5 years, *N* = 434	Maximal progressive treadmill run to assess *V*O_2max_ in ml/min/kg, divided into quartiles	Clustered risk (sum of z-scores > 1SD) including TC:HDL, TG, SBP, HOMA-IR and SumSF	Not specified	**Clustered risk** Quartile 1: OR 6.8 (95% CI 2.2; 21.0) Quartile 2: OR 2.9 (95% CI 0.9; 9.5) NS Quartile 3: OR 3.3 (95% CI 1.0; 10.5) Compared to upper fitness quartile
Barnekow-Bergkvist et al. (2001) [55]	*N* = 425, 52%boys, 16.1 ± 0.33 years (range 15–18 years), Sweden	18 years, *N* = 278	9-min run/walk test, distance covered in meters, 9-min run (M ≥ 2,150 m F ≥ 1614 m) for Relative Risk and for logistic regression (OR) each 100-m decrease	BMI (M ≥ 27; F ≥ 27)	Sport club membership, Satisfied with sports performance, positive attitude to soccer, handball and aerobic exercise, BMI, School program level	**BMI** Bivariate RR: (M) 0.9 (95% CI 0.4; 1.9) NS; (F) 0.5 (95% CI 0.2; 1.2) NS Multiple logistic: (M) OR 1,4 (95% CI 1.1; 1.9); (F) OR 1.0 (95% CI 0.7; 1.5) NS
				WHR (M ≥ 0.95; F ≥ 0.85)		
						**WHR** Bivariate RR: (M) 0.4 (95% CI 0.1; 1.3) NS; (F) OR 0.5 (95% CI 0.2; 1.2) NS Multiple Logistic: (M) OR 1.3 (95% CI 0.9; 1.8) NS; (F) N/A
				SBP ≥ 140 mmHg		**SBP** Bivariate RR: (M) 1.1 (95% CI 0.6; 2.0) NS; (F) 1.0 (95% CI 0.3; 3.2) NS OR: N/A
				TC (M ≥ 6.2; F ≥ 6.2)		**TC** Bivariate RR: (M)1.0 (95% CI 0.5; 2.0); (F) 0.5 (95% CI 0.2; 1.3) OR: N/A
Byrd-Williams et al. (2008) [65]	*N* = 160, 53% boys, age boys 11.2 ± 1.6, girls 11.2 ± 1.8 years (range 8–13), USA (only Hispanic overweight children)	4 years, *N* = 160	Maximal progressive treadmill test to assess *V*O_2max_ in ml/min and ml/min/kg	Change in total fat mass (kg) over age	Changes in total lean tissue mass, Tanner stage, sex and age	**Fat mass** (M) *B* = − 0.001 (SE 0.0004) *P* < 0.05 (F) *B* = 0.0005 (SE 0.0005) NS
Chen et al. (2014) [40]	*N* = 2758, 51%boys, age 9.7 ± 0.5 years, Taiwan	2 years, *N* = 1933	800-m sprint test time z-scores based on sex and age specific means and SD; a positive z-scores indicates high fitness	WC ≥ 85%, WHR ≥ 85%, WHtR ≥ 85%	Age, sex, parental educational level, family income, family history of atopy, breastfeeding, maternal smoking in pregnancy	**WC:** OR 1.14 (95% CI 1.12; 1.16) **WHR:** OR 1.11 (95% CI 1.09; 1.14) **WHtR:** OR 1.13 (95% CI 1.11; 1.16)
Dwyer et al. (2009) [66]	*N* = 8498 of whom *N* = 2595 with CRF, 51% boys, age boys 11.9 ± 2.4, girls 11.8 ± 2.4 years (range 7–15 years), Australia	19–21 years, *N* = 647	Bicycle ergometer to assess physical working capacity at HR 170 bpm, as watts per kg lean mass expressed as unfit vs. normal fitness	BMI, Obesity	Sex, age, SES at baseline, and education level at follow-up. Obesity status and BMI additionally adjusted for BMI at baseline.	**BMI** *B* = 0.96 (95% CI 0.34; 1.58) **Obesity** OR 3.0 (95% CI 1.5; 5.6)
				Insulin resistance (= HOMA-IR ≥ 75th sex-specific percentile) HOMA-IR		**Insulin resistance** OR 1.7 (95% CI 1.1; 2.6) **HOMA-IR** OR 0.18 (95% CI − 0.0003; 0.36) NS
Eisenmann et al. (2005) [33]	*N* = 48, 75% boys, age boys: 15.9 ± 1.9, girls 15.2 (± 2.5) years, USA (only those < 18 years old measured)	15 years, *N* = 48	Maximal treadmill test, modified Balke protocol. Expressed as treadmill time	BMI	Length of follow-up, sex and age	**BMI** *r* = − 0.34 (*P* < 0.05) Low vs. high fitness: 24.6 (3.2) vs. 22.9 (2.4) (*P* < 0.05)
						**WC** *r* = − 0.38 (*P* < 0.05) Low vs. high fitness: 84.5 (11.4) vs. 79.6 (9.3) (*P* < 0.05) **%BF** *r* = − 0.47 (*P* < 0.05) Low vs. high fitness: 19.9 (4.9) vs. 14.6 (6.5) (*P* < 0.05)
				WC %BF		
				SBP, DBP, MAP		
				TC, HDL-C, TC:HDL, TG		**SBP** *r* = − 0.01 NS; Low vs. high fitness: 117.5 ± 9.8 vs. 116.9 ± 12.6 NS **DBP** *r* = − 0.12 NS; low vs. high fitness: 77.9 ± 7.6 vs. 77.1 ± 11.2 NS **MAP** *r* = − 0.10 NS; low vs. high fitness MAP 91.1 ± 6.7 vs. 90.4 ± 10.8 NS
				Glucose		
				Composite metabolic risks score		
						**TC** *r* = − 0.20 NS; Low vs. high fitness: 189.8 ± 44.6 vs. 184.3 ± 42.4 NS **HDL-C** *r* = − 0.15 NS; Low vs. high fitness: 52.1 ± 17.3 vs. 44.9 ± 9.8 NS **TC:HDL** *r* = − 0.08 NS; Low vs. high fitness: 4.0 ± 1.6 vs. 4.3 ± 1.4 NS **TG** *r* = 0.03 NS; Low vs. high fitness: TG 96.7 ± 68.1 vs. 123 ± 67.9 NS
						**Glucose** *r* = 0.12 NS 4.0 (1.6) vs. 4.3 (1.4) NS: 93.2 ± 7.5 vs. 93.3 ± 6.0 NS
						Composite metabolic risk score *r* = 0.03 NS
Ekblom et al. (2009) [41]	*N* = 508, 56% boys, age 10 years, Sweden	6 years, *N* = 296	Submaximal ergometer test with estimated maximal VO2 in ml/min/kg—high fitness (top 2 tertiles) vs. low fitness	High BMIsds (> 2 sds), and increasing BMIsds (BMIsds-difference > 0)	Sex, PE teacher education level, level of MVPA, geographic region of school, BMIsds baseline	**High BMIsds:** OR 0.13 (95% CI 0.04; 0.44) **Increasing BMIsds**: no values given, NS
Flouris et al. (2008) [67]	*N* = 210, 56% boys, age boys 12.3 ± 0.6, girls 12.3 ± 0.6 years, Greece	6 years, *N* = 203	20-m MSRT, calculated *V*O_2max_ in ml/min/kg	Metabolic syndrome (≥ 3 of 5 symptoms); SBP ≥ 90th (age, height and sex specific); HDL-C (M) < 45 mg/dl (F) < 50 mg/dl; TG ≥ 150 mg/dl; glucose ≥ 110 mg/dl; BMI ≥ 90th	Not specified	**MetS** Cut off point of *V*O_2max_ for prediction of metabolic syndrome (Z value for Cohen’s Kappa): (M) 12yrs 31.8 (3.424), 13yrs 37.5 (3.341), 14yrs 37.8 (2.344); (F) 12yrs 26.8 (2.290), 13yrs 28.3 (3.341). 14yrs 28.3 (1.750) NS
Freitas et al. (2012) [68]	*N* = 450, 51% boys, age group 1 (8 years), group 2 (12 years) and group 3 (16 years), Portugal	7.2 years, *N* = 434	12-min run/walk test, distance covered	BMI	Not specified	NP means that fitness was not a predictor and it was not included in the model, therefore no data available. **BMI** (M) group 1: NP, group 2 NP, group 3 NP (F) group 1 NP, group 2 NP, group 3 NP
				WC		**WC** (M) group 1: NP, Group 2 NP, group 3 NP (F) group 1 NP, group 2 NP, group 3 NP
				SumSF		**SumSF** (M) group 1: NP, Group 2 NP, group 3 *B* = − 0.014 Partial R^2^ = 0.03, *P* < 0.05 (F) group 1 NP, group 2 NP, group 3 NP
Hasselstrom et al. (2002) [60]	*N* = 305, 44% boys, age 17.1 ± 1.0 years (range 15–19 years), Denmark	8 years, *N* = 203	Maximal progressive cycle ergometer test, measured *V*O_2max_ in ml/min/kg	WC	Age	**WC**: (M) *B* = 0.08 *r* = − 0.10 NS; (F) *B* = − 0.06 *r* = − 0.08 NS
				%BF		**%BF:** (M) *B* = − 0.22 *r* = − 0.18 NS, (F) *B* = − 0.38 *r* = − 0.27 *P* < 0.05
				SBP, DBP		**SBP**: (M) *B* = 0.05 *r* = 0.097 NS, (F) *B* = − 0.02 *r* = − 0.04 NS **DBP**: (M) *B* = − 0.61 *r* = − 0.11 NS, (F) *B* = 0.03 *r* = − 0.04 NS
				TC, HDL-C, TC:HDL, TG		
				Metabolic risk score risk score calculated as the sum of SBP, TC, TC:HDL, TG, and %BF (from skinfolds)		**TC**: (M) *B* = − 0.25 *r* = − 0.04 NS, (F) *B* = − 0.93 *r* = − 0.18 NS; **HDL-C**: (M) *B* = − 2.16 *r* = − 0.09 NS, (F) *B* = 0.72 *r* = 0.04 NS **TC:HDL** (M) *B* = − 5.84 *r* = − 0.07 NS, (F) *B* = 13.43 *r* = 0.19 NS **TG** (M) *B* = 1.68 *r* = 0.14 NS, (F) *B* = − 5.16 *r* = − 0.24, *P* < 0.05
						**Metabolic risk score**: (M) *B* = − 0.25 *r* = − 0.18 NS, (F) *B* = − 0.01 *r* = 0.00 NS
Henderson et al. (2016) [43]	*N* = 630, 54.4% boys, age 9.6 ± 0.9 years, Canada (white, ≥ one obese parent)	2 years, *N* = 564	Maximal progressive cycle ergometer test, measured VO2peak as ml/min/FFM	Insulin sensitivity by Matsuda index; HOMA-IR	1. Crude 2. Sex, age, Tanner stage, season 3. Also %BF	**Insulin sensitivity:** 1) *B* = 0.6 (95% CI − 0.5; 1.7) NS 2) *B* = − 0.05 (95% CI − 1.1; 1.0) NS 3) *B* = 0.3 (95% CI − 0.6; 1.1) NS **HOMA-IR:** 1) *B* = − 0.2 (95% CI − 1.4; 0.9) NS 2) *B* = 0.4 (95% CI − 0.7; 1.5) NS 3) *B* = − 0.05 (95% CI − 0.9; 0.9) NS
Janz et al. (2000) [44]	*N* = 123, 50% boys, age boys 10.8 ± 1.0, girls: 10.3 ± 1.0 years (range 7–12 years), USA	5 years, *N* = 110	Maximal progressive ergometer test. Peak *V*O_2max_ in ml/min	LVM; change in LVM	Age, FFM, height, peak SBP, SBP, SumSF, peak *V*O_2max_ and maturity (Tanner and for boys testosterone) All predictor variables were included in the model and deleted if *P* > 0.05	**LVM** Spearman correlation (M) *r* = 0.5; (F) *r* = 0.54 **LVM** (M) only FFM in model; (F) peak oxygen uptake explained ± 35%, and when FFM was added it explained 43% of the variability in LVM together
Johnson et al. (2000) [45]	*N* = 115, 31% boys, age white boys 8.7 (± 1.76); black boys 7.6 (± 1.5); white girls 8.1 (± 1.38); black girls 8.1 (± 1.73) years, USA	5 years, *N* = 95	Maximal progressive walking treadmill test to measure *V*O_2max_ in L/min	The increase in fat mass adjusted for the increase in lean mass. (FM/FFM)	Initial FM, LTM, and age Tanner stage, ethnicity and baseline energy expenditure	**FM/FFM** *B* = − 2, *P* = 0.05
Klakk et al. (2014) [46]	*N* = 800, 44%boys, age 9.4 ± 0.8 years (range 7.7–11.4 years), Denmark	2 years, *N* = 365	Andersen Test, 10-min intermittent running test in meters	SBP	Model 1: baseline values of risk, age, sex, school type, pubertal status, birth weight, and parental educational level (WC also height and height^2^) Model 2: also BF%	**SBP** 1) *β* − 0.05 (95% CI − 0.14; 0.04) NS; 2) *β* − 0.05 (95% CI − 0.05; 0.15) NS
				TC:HDL, TG		**TC:HDL** 1) *β* − 0.06 (95% CI − 0.12; 0.01) NS; 2) *β* − 0.02 (95% CI − 0.10; 0.06) NS **TG** 1) *β* − 0.09 (95% CI − 0.19; 0.01) NS; 2) *β* − 0.02 (95% CI − 0.13; 0.10) NS
				HOMA-IR		
				Composite Risk score: standardized scores of logHOMA-IR, SBP, logTC:HDL and logTG		**HOMA-IR** 1) *β* − 0.16 (95% CI − 0.27; − 0.05); 2) *β* − 0.03 (95% CI − 0.14; 0.09) NS
						**Composite risk score** 1) *β* − 0.12 (95% CI − 0.21; − 0.02); 2) *β* − 0.009 (95% CI − 0.11; 0.09) NS
Latt et al. (2016) [70]	*N* = 313, 100% boys, age 11.9 ± 0.1 years, Estonia	2 years, *N* = 120	Maximal progressive cycle ergometer test, measured *V*O_2max_ in ml/min/kg or per FFM. Low < 45 per kg or < 65.3 per FFM. Moderate 45–53 per kg or 65.3–71.7 per FFM High > 53 per kg or > 71.7 per FFM	TC:HDL, TG HOMA-IR	Tanner stage and second-year follow-up CRF	**TC:HDL** Low vs. High *V*O_2max_/kg OR 3.49 (95% CI 1.23; 9.86) Low vs. High *V*O_2max_/FFM OR 0.77 (95% CI 0.31; 1.97) NS **TG** Low vs. High *V*O_2max_/kg OR 2.99 (95% CI 1.07; 8.38) Low vs. High *V*O_2max_/FFM OR 1.15 (95% CI 0.47; 2.82) NS
						**HOMA-IR:** Low vs. High *V*O_2max_/kg OR 5.93 (95% CI 2.01; 13.38) Low vs. High *V*O_2max_/FFM OR 1.23 (95% CI 0.50; 3.03) NS
Liew et al. (2011) [47]	*N* = 533, 54% boys, age 10.9 ± 0.49 years, USA	4 years, *N* = 246	1 mile walk/run time	BMI	Age and sex	**BMI** Partial correlation coefficients: 5th grade with 8th grade: *r* = 0.21 NS; 7th grade: *r* = 0.25 *P* < 0.05, 6th grade with 8th grade BMI: *r* = 0.56 *P* < 0.01
Lopes et al. (2012) [37]	*N* = 285, 50% boys, age boys 5.9 ± 0.3, girls 5.9 ± 0.3 years, Portugal	4 years, *N* = 285	1 mile walk/run time	SumSF	Sex, time squared, time cubed, motor coordination, curl-up, push-up, baseline SumSF	**SumSF** *B* = 0.12 (SE 0.05) (95% CI 0.02; 0.22)
Martins et al. (2009) [48]	*N* = 153, 43% boys, age boys 9.1 ± 0.87, girls 9.1 ± 0.90 years, Portugal	5 years, *N* = 153	20-m MSRT, estimated *V*O_2max_ in ml/min/kg	BMI	Model 1 for time Model 2 also for sex and age	**BMI** 1) *B* = − 0.14 (95% CI − 0.20; − 0.09), 2) *B* = − 0.15 (95% CI − 0.21; − 0.09)
				SBP, DBP		**SBP** 1) *B* = − 0.04 (95% CI − 0.18; 0.25) NS, 2) *B* = 0.04 (95% CI − 0.18; 0.27) NS **DBP** 1) *B* = − 0.13 (95% CI − 0.33; 0.66) NS, 2) *B* = − 0.02 (95% CI − 0.22; − 0.19) NS
				TC		**TC** 1) 0 = B− 0.46 (95% CI − 1.02; 0.95) NS, 2) *B* = − 0.18 (95% CI − 0.77; − 0.41) NS
McGavock et al. (2009) [49]	*N* = 222, =%boys N/A, age 11 years, Canada	2 years, *N* = 222	20-m MSRT, estimated *V*O_2max_ in ml/min/kg	BMI	Age, baseline BMI and sex	**BMI** *B* = − 0.09 (SE 0.05) NS
McMurray et al. (2008) [50]	*N* = 2207, 55% boys, age 8.6 ± 0.8 years, USA	6.5 years, *N *= 389	Multi-stage submaximal cycle ergometer test to estimate *V*O_2max_, *V*O_2max_ in ml/min/kg or in ml/min/FFM in tertiles	Presence of MetS (criteria of Jolliffe and Janssen)	Sex, baseline BMI and blood pressure (both > sex and age specific 95th percentile), cholesterol (> 200 mg/dl)	**MetS** Low vs. high *V*O_2max_/kg: OR 6.09 (95% CI 1.184; 60.296) Low vs. moderate *V*O_2max_/kg: OR 5.58 (95% CI 1.152; 53.775) Low vs. high *V*O_2max_/FFM: OR 3.64 (95% CI 0.93; 20.826) Low vs. moderate *V*O_2max_/FFM: OR 5.71(95% CI 1.197; 54.455)
Mikkelsson et al. (2005) [35]	*N* = 624, 100% boys, age 12–17 years, Finland	25 years, *N* = 29 from subgroup with clinical assessment	2000-m distance run test, classified as slow and fast runners based on a median split per age group	SBP, DBP	In ANCOVA 1. For age and 2. Also for adult BMI	**SBP** 1) 141 mmHg (95% CI 134; 148) vs. 134 mmHg (95% CI 126; 141) NS 2) *P* = 0.05 **DBP** 1) 90 mmHg (95% CI 86; 93) vs. 83 mmHg (95% CI 80; 87), *P* = 0.013; 2) *P* = 0.003
Ortega et al. (2011) [51]	*N* = 1144, 46% boys, age 9.5 ± 0.4 years, Estonia and Sweden	6 years, *N* = 598	Maximal progressive cycle ergometer test, estimated *V*O_2max_ in ml/min/kg	Incidence of overweight/obesity at follow-up for normal weight children at baseline	1. Country, sex, age, and sexual maturation 2. Baseline BMI	**Overweight/obesity incidence** 1) OR 0.89 (95% CI 0.84; 0.95). 2) OR 0.96 (95% CI 0.89; 1.04) NS
Savva et al. (2014) [36]	*N* = 4878, 46%boys, age boys 11.4 ± 0.4, girls 11.4 ± 0.3 years. Range 10–13.5 years, Cyprus	4.6 years, *N* = 4878	20-m MSRT, estimated *V*O_2max_ in ml/min/kg divided into quartiles	Incidence of overweight/obesity according to IOTF criteria	1. Unadjusted 2. Age, length of follow-up, area of residence, and average monthly air temperature at time of fitness test	**Overweight/obesity** 1) (M) Quartile 2: OR 0.82 (95% CI 0.55; 1.23) NS; Quartile 3: OR 0.66 (95% CI 0.44; 0.99); Quartile 4: OR 0.40 (95% CI 0.26; 0.61) (F) Quartile 2: OR1.23 (95% CI 0.76; 2.02) NS; Quartile 3: OR 0.50 (95% CI 0.28; 0.88); Quartile 4: OR 0.55 (95% CI 0.32; 0.95) 2) (M) Quartile 2: OR 0.85 (95% CI 0.5; 1.28) NS; Quartile 3: OR 0.68 (95% CI 0.46; 1.03) NS; Quartile 4: OR 0.40 (95% CI 0.26; 0.61) (F) Quartile 2: OR1.26 (95% CI 0.77; 2.08) NS; Quartile 3: OR 0.52 (95% CI 0.29; 0.92); Quartile 4: OR 0.57 (95% CI 0.33; 0.99)
Schmidt et al. (2016) [69]	*N* = 8498, % boys unknown, age 7–15 years, Australia	19.9 years, *N* = 1792 (52.3% boys)	1 mile run, run time used to estimate *V*O_2max_, in tertiles (< 20th percentile (low), 20–59th percentile (moderate) and ⩾60th percentile (high))	Metabolic syndrome (≥ 3:WC (M) ≥ 102 cm (F) ≥ 88 cm; SB ≥ 130 mmHg or DBP ≥ 85 mmHg or treatment; HDL-C (M) < 1.0 mmol/, (F) < 1.29 mmol/L or treatment; TG ≥ 1.70 mmol/L or treatment; glucose ≥ 5.6 mmol/L or treatment	1. Age, sex, length of follow-up 2. Also for waist circumference	**MetS** 1) Mid fitness: RR 0.55 (95% CI 0.37; 0.80). High fitness RR 0.46 (95% CI 0.30; 0.69). 2) Mid fitness: RR 0.68 (95% CI 0.46; 1.01) NS. High fitness RR 0.64 (95% CI 0.43; 0.96)
Sun et al. (2014) [34]	*N* = 8498, 51% boys, age boys 11.2 ± 2.5 girls 10.9 ± 2.6 years, Australia	20 years, *N* = 1976	1.6-km run, inverse of time to complete	Serum hsCRP and plasma fibrinogen	1. Age, childhood and adulthood SES, smoking, fat intake and alcohol consumption, education, and hormonal contraceptive use for females 2. Also adiposity	**hsCRP** (M)Crude: *B* = − 0.11 (95% CI − 0.19; − 0.03) *P* = 0.005 (F) *B* = − 0.22 (95% CI − 0.3; − 0.14) *P* < 0.001 1) *B* = − 0.11(95% CI − 0.19; − 0.03) *P* = 0.005 (F) *B* = − 0.24 (95% CI − 0.32; − 0.17) *P* < 0.001 2) *B* = − 0.07 (95% CI − 0.15; 0.01) NS (F) *B* = − 0.20 (95% CI − 0.28; − 0.12) *P* < 0.001 **Fibrinogen** (M) Crude: *B* = − 0.13 (95% CI − 0.17; − 0.09) *P* < 0.001 (F) *B* = − 0.13 (95% CI − 0.17; − 0.08) *P* < 0.001 1) *B* = − 0.13 (95% CI − 0.17; − 0.09) *P* < 0.001 (F) *B* = − 0.14 (95% CI − 0.19; − 0.09) *P* < 0.001 2): *B* = − 0.11 (95% CI − 0.14; − 0.07) *P* < 0.001 (F) *B* = − 0.10 (95% CI − 0.15; − 0.05) *P* < 0.001
Telford et al. (2015) [52]	*N* = 694, 50% boys, age 8.1 (± 0.3) years	4 years, *N* = 469	20-m MSRT, number of stages	HDL-C, HDL-C and logTG	1. Height, age, pubertal development, school, and socioeconomic status 2. Also BF% in case model 1 was significant	**HDL-C** 1) (M) *B* = − 0.17 (SE 0.07 Effect size: − 2.4; *P* = 0.014), 2) no values given NS 1) (F) *B* = − 0.09 (SE 0.085 Effect size 0.22) NS **HDL-C** 1) (M) *B* = − 0.003 (SE 0.035 Effect size 0.09) NS 1) (F) *B* = − 0.02 (SE 0.04 Effect size 0.05) NS **LogTG** 1) (M) *B* = − 0.14 (SE:0.063; Effect size − 2.2; *P* = 0.03), 2) NS 1) (F) *B* = − 0.14 (SE:.07; Effect size: − 2.0; *P* = 0.04), 2) NS
Treuth et al. (2003) [53]	*N* = 101, 0% boys, age 8–9 years, USA	2 years, *N* = 88	Maximal progressive treadmill test measured VO2peak in ml/min	FM and %BF	Time, ethnicity, Tanner stage, parent weight group, baseline weight	**FM** Estimate − 0.004 (SE 0.001) *P* < 0.01 **%BF** Estimate − 0.008 (SE 0.0028) *P* = 0.008
Yoonsuk et al. (2014) [62]	*N* = 1006, 59%boys, age 17 years, Korea	23 years, *N* = 1006	100-m dash time (s), standing long jump distance (cm), sit and reach distance (flexibility test, cm), 1000-m (male)/800-m (female) meter (min), sit-ups (reps) and chin-ups (male) or arm-hanging (female). Each test was converted to categorical scale and sum scores were divided in tertiles	BMI ≥ 25 kg/m^2^	Not specified	**BMI** (M) Mid: OR 1.6 (95% CI 0.99; 2.57) NS; Low: OR 2.23 (95% CI 0.76; 6.52) NS. (F) Mid: OR 1.40 (95% CI 0.78; 2.53) NS; Low: OR 2.48 (95% CI 0.99; 6.23) NS
				WC ≥ 90 cm		
						**WC** (M) Mid: OR 1.18 (95% CI 0.75; 1.85) NS; Low: OR 1.55 (95% CI 0.89; 2.68) NS. (F) Mid: OR 1.26 (95% CI 0.77;2.06) NS; Low: OR 2.34 (95% CI 1.03;5.32)
				SBP ≥ 130 mmHg or DBP ≥ 85 mmHg		
						**SBP or DBP** (M) Mid: OR 1.12 (95% CI 0.78; 1.62) NS; Low: OR 0.90 (95% CI 0.41; 1.99) NS. (F) Mid: OR 1.15 (95% CI 0.63; 2.11) NS; Low: OR 1.77 (95% CI 0.66; 4.70) NS
				HDL-C < 40 mg/dl, TG ≥ 150 mg/d		
						**HDL-C** (M) Mid: OR 0.88 (95% CI 0.56; 1.38) NS; Low: OR 1.38 (95% CI 0.58; 3.24) NS. (F) Mid: 1.44 (95% CI 0.87; 2.39) NS; Low: 2.34 (95% CI 1.02; 5.41) NS **TG** (M) Mid: OR 1.04 (95% CI 0.74; 1.47) NS; Low: OR 0.73 (95% CI 0.35; 1.52) NS. (F) Mid: 1.31 (95% CI 0.65; 2.65) NS; Low: 1.42 (95% CI 0.43; 4.75) NS
				Fasting glucose ≥ 110 mg/dl		
						**Glucose** (M) Mid: OR 0.89 (95% CI 0.52; 1.53) NS; Low: OR 0.68 (95% CI 0.19; 2.40) NS. (F) Mid: 0.60 (95% CI 0.18; 2.01) NS; Low: 0.81 (95% CI 0.09; 7.22) NS
				MetS 1 or more of above risk factors		
						**MetS** (M) Mid: OR 0.88 (95% CI 0.55; 1.41) NS; Low: OR 0.89 (95% CI 0.33; 2.37) NS. (F) Mid: N/A; Low: N/A

*β* standardized regression coefficient, *B* unstandardized regression coefficient, *OR* odds ratio, *RR* relative risk, *N/A* not available, *M* male, *F* female, *BMI* body mass index, *BMIc* BMI corrected for age and sex, *WC* waist circumference, *WHR* waist-hip ratio, *WHtR* waist-to-height ratio, *FM* fat mass, *FFM* fat free mass, *%BF* body fat percentage, *SumSF* Sum of skinfolds, *SBP* systolic blood pressure, *DBP* diastolic blood pressure, *MAP* mean arterial pressure, *NS* not significant, *TC* total cholesterol, *HDL*-*C* high density lipoprotein-cholesterol, *LDL*-*C* low density lipoprotein-cholesterol, *TC:HDL* ratio of total cholesterol and high density lipoprotein-cholesterol, *TG* triglycerides, *HOMA*-*IR* homeostatic model assessment of insulin resistance, *HOMA*-*B* homeostatic model assessment of beta cell function, *HbA1c* glycated hemoglobin, *hsCRP* high sensitivity c-reactive protein, *IMT* intima media thickness, *LVM* left ventricular mass, *MetS* metabolic syndrome, *IOTF* International Obesity Task Force

**Table 3:**
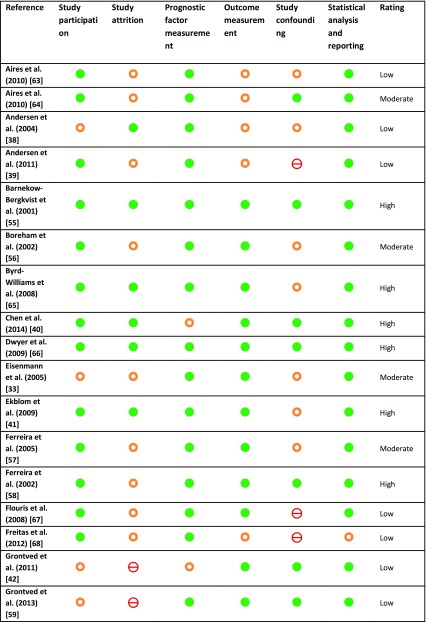

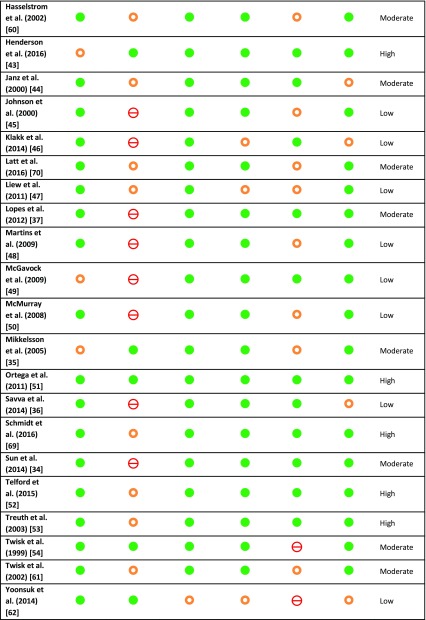
Risk of bias assessment and overall quality rating based on STROBE and QUIPS checklists

low risk of bias,  moderate risk of bias,  high risk of bias

*QUIPS* Quality in Prognostic Studies, *STROBE* Strengthening the Reporting of Observational Studies in Epidemiology

**Table 4:** Summary of the association between childhood CRF and CVD risk outcomes

Sex	Association	References
**Body mass index/weight status**		
Boys	**[+]** [o] [+] [o]	**[**55**]** [68] [36] [62]
Girls	**[o]** [o] [+] [o]	**[**55**]** [68] [36] [62]
Not separately reported	[o] [+] **[+]** [+] **[+]** [+^2yr^/o^3yr^] [+] [o] **[o]**	[63] [64] **[**66**]** [33] **[**41**]** [47] [48] [49] **[**51**]**
**Waist circumference**		
Boys	[o] [o] [o]	[60] [61] [62]
Girls	[o] [o^absolute 13yr^/+^absolute 13–16yr^/+^kg 13yr^/o^kg 13–16yr^] [+]	[60] [61] [62]
Not separately reported	**[+]** [+] [o]	**[**40**]** [33] [68]
**Waist-to-hip ratio**		
Boys	**[o]**	**[** 55 **]**
Girls	**[o]**	**[** 55 **]**
Not separately reported	**[+]** [o]	**[**40**]** [61]
**Body composition**		
Boys	[+] **[+]** [o^8&12yr^/+ ^16yr^] [o] [+]	[56] **[**65**]** [68] [60] [54]
Girls	[+] **[o]** [o] [+] [+]	[56] **[**65**]** [68] [60] [54]
Not separately reported	[+] [+] [+] **[+**^**FM&%BF**^**]** [o^absolute^/+^kg^]	[33] [45] [37] **[**53**]** [61]
**Systolic blood pressure**		
Boys	**[o]** [o] [o] [+] [+^absolute 13yr^/o] [o]	**[**55**]** [56] [60] [35] [61] [62]
Girls	**[o]** [o] [o] [o] [o]	**[**55**]** [56] [60] [61] [62]
Not separately reported	[o] [o/+^RPP^] [o] [o]	[33] [42] [46] [48]
**Diastolic blood pressure**		
Boys	[o] [o] [+] [+] [o]	[56] [60] [35] [54] [62]
Girls	[o] [o] [o] [o]	[56] [60] [54] [62]
Not separately reported	[o] [o] [o]	[33] [48] [61]
**Total cholesterol**		
Boys	**[o]** [o] [o]	**[**55**]** [56] [60]
Girls	**[o]** [o] [o]	**[**55**]** [56] [60]
Not separately reported	[o] [o] [o]	[33] [48] [61]
**HDL-Cholesterol**		
Boys	[o] [o] [o]	[56] [60] [62]
Girls	[o] [o] [o]	[56] [60] [62]
Not separately reported	[o] **[o]** [o]	[33] **[**52**]** [61]
**TC:HDL ratio**		
Boys	[o^12yr^/+^15yr^] [o] [o] [+^kg^/o^FFM^] [+]	[56] [60] [46] [70] [54]
Girls	[+^12yr^/o^15yr^] [o] [o] [+]	[56] [60] [46] [54]
Not separately reported	[o] [o]	[33] [61]
**Triglycerides**		
Boys	[o] [+^kg^/o^FFM^] **[o]** [o]	[60] [70] **[**52**]** [62]
Girls	[+] **[o]** [o]	[60] **[**52**]** [62]
Not separately reported	[o] [o]	[33] [46]
**Glucose**		
Boys	[o]	[62]
Girls	[o]	[62]
Not separately reported	[o] [o]	[33] [59]
**HOMA-IR**		
Boys		
Girls		
Not separately reported	**[o/+**^**75th**^**]** [+] **[o]** [o] [+^kg^/o^FFM^]	**[**66**]** [59] **[**43**]** [46] [70]
**Metabolic syndrome or composite risk scores**		
Boys	[+] [o]	[67] [62]
Girls	[+/o^14yr^]	[67]
Not separately reported	[o] [+] [o] [o] [o] [o] [+] **[+]**	[38] [39] [33] [57] [60] [46] [50] **[**69**]**

Each association is represented as [+] indicating better outcome with high baseline fitness, [−] indicating poorer outcome with high baseline fitness, [o] indicating no significant association. Bold indicates high-quality article

Superscript indicates different associations within article (sub)groups: 2 yr = 2 years of follow-up; 3 yr = 3 years of follow-up; absolute 13 yr = CRF expressed in absolute values at age 13; absolute 13–16 yr = CRF expressed in absolute values and averaged between 13 and 16 years; kg 13 yr = CRF expressed per kg of body mass at age 13; kg 13–16 yr = CRF expressed per kg of body mass and averaged between 13 and 16 years; 8&12 yr = CRF at age 8 and 12 years; 16 yr = CRF at age 16 years; FM&%BF = both fat mass and  %body fat as outcome; absolute = CRF expressed in absolute values; kg = CRF expressed per kg body mass; RPP = CRF expressed by rate pressure product; 12 yr = CRF at age 12 years; 15 yr = CRF at age 15 years; FFM = CRF expressed per kg fat free mass; 75th = outcome cut-off above 75th percentile; 14 yr = CRF at age 14

*CRF* cardiorespiratory fitness, *CVD* cardiovascular disease

## Results

The literature search yielded a total of 9158 references (Fig. 1). After removal of duplicates there were 7524 unique references, and by screening the titles and abstracts we excluded 7178 references. Of the remaining 346 articles, 96 full texts of articles were neither retrievable from the libraries of our institutes nor from the authors. The other 173 articles were excluded due to reasons indicated in Fig. 1, most because of determinants or outcomes that were irrelevant to our aim. Eventually, 38 articles met the inclusion criteria and were used for further analyses.

### Study Characteristics

All included articles reported on prospective cohorts, and there were no randomized controlled trials (RCT). A summary of the key characteristics of the included articles and the outcomes of interest is presented in Table 2.

The median number of included children and adolescents per article at baseline was *N* = 479, ranging from *N* = 48 [33] up to *N* = 8498 [34]. At follow-up the median number of included participants was *N* = 291, ranging from *N* = 29 [35] to *N* = 4878 [36]. Mean age at baseline of the participants of the included articles ranged from 5.9 years [37] up to 17.5 years old [38], with a median age of 11.3 years. Most articles studied pre-adolescen t children at baseline, i.e., younger than 12 years [37, 39–54]. In 11 articles the mean age of the included participants at baseline was 12 years or older; these children were considered adolescents [33, 35, 38, 55–62]. In the ten remaining articles both children and adolescents were included [34, 36, 63–70]. The median follow-up time was 6 years, varying from 2 years [40, 43, 46, 49, 53, 64, 70] to 25 years [35].

Physical fitness was measured in a variety of ways (Table 2). Twenty-one articles (55%) reported measured or estimated *V*O_2max_ or peak oxygen uptake, either expressed as absolute values or adjusted for body mass or FFM. Of these, 11 articles reported objectively measured CRF by using a maximal exercise test with direct assessment of *V*O_2max_ or peak oxygen uptake [33, 38, 39, 42–45, 51, 53, 57–61, 65, 70], while others used submaximal or field tests to estimate *V*O_2max_. Six articles reported the time it took to complete a certain test [33–35, 37, 40, 47]. The achieved level of the 20-m Multi Stage Shuttle Run Test (20 m-MSRT) was used in three articles [52, 54, 56]. Three articles used the distance covered within a pre-specified time [46, 55, 68]. A composite score of multiple health-related fitness components was used in three articles [62–64]. Furthermore, physical working capacity on an ergometer at heart rate 170 was used [66]. One article used various hemodynamic properties during a graded maximal fitness test [42]. CRF was expressed as a continuous measure in most articles, but in 11 articles authors categorized CRF using different cut-offs [35, 36, 38, 41, 50, 54, 62–64, 66, 70].

Some of the included articles reported on the same cohort; these included two articles about a cohort of Portuguese schoolchildren [63, 64]; two articles based on the Northern Ireland Young Hearts Project [54, 56]; three from the Amsterdam Growth and Health Longitudinal study [57, 58, 61]; and two based on the European Youth Heart Study [42, 59]. These articles are shown first in Table 2. We assessed these articles separately, since they included different subsamples or measured different outcomes at different times, but the reader should be aware that the included samples in these articles could overlap.

### Quality Assessment

The methodical quality of articles was assessed by the STROBE and QUIPS checklists, and results are presented in Table 3. Overall, 11 articles were qualified as high quality [40, 41, 43, 51–53, 55, 58, 65, 66, 69], and 12 articles were of moderate quality [33–35, 37, 44, 54, 56, 57, 60, 61, 64, 70], which was in most cases due to unclear attrition. The remaining 15 articles were judged to be of low overall quality. The most common issue was the absence of clear descriptions about study attrition and lack of or unclear description about adjustments for confounding in analyses.

### Association Between Childhood Baseline Physical Fitness and CVD Risk Factors

A summary of the associations between childhood baseline CRF and the different CVD risk factors at least 2 years later is presented in Table 4. A [+] indicates a significant association between higher baseline fitness and better health outcomes, e.g., a negative regression coefficient indicating higher fitness was associated with lower BMI. A [−] indicates a significant association between higher baseline fitness and poorer health outcomes, e.g., a positive regression coefficient indicating higher fitness was associated with higher BMI. Articles with no significant association are presented as [o]. High-quality articles are indicated by bold print in Table 4. We only included the fully adjusted models when there were multiple models reported in an article. All significant associations were in the expected direction, and most of the non-significant associations were also in the expected direction.

### BMI

In boys, higher CRF was associated with healthier (lower) BMI in two out of four articles [36, 55], of which one was of high quality [55]. In girls, only Savva et al. showed that higher CRF was associated with a lower risk of being overweight [36]. Three other articles, including one high-quality article [55], showed no associations. Five articles, of which two were of high quality, found that better CRF was associated with lower BMI in both sexes combined [33, 41, 48, 64, 66], while three other articles, of which one was of high quality [51], found no associations [49, 63]. Liew et al. showed a significant association between CRF and BMI for children aged 11 years after 2 years’ follow-up, which was no longer significant at the third year of follow-up [47]. Of the three articles reporting on adolescents [33, 55, 62], higher CRF was associated with lower BMI in boys [55] and both sexes combined [33], while the other article showed no association [62]. Only one article used objectively measured *V*O_2max_, which was associated with lower BMI [33].

### Waist or Hip Measurements

In boys, none of the articles reported a significant association between childhood CRF and waist circumference (WC) [60–62]. In girls, Yoonsuk et al. reported higher odds of increased WC for those in the lowest tertile of an aggregated fitness test [62]. Twisk et al. measured CRF by *V*O_2max_ as absolute measure and per kg of body mass, and CRF was assessed at baseline (age 13 years) or as the average CRF between age 13 and 16 years. Girls with higher absolute *V*O_2max_ between 13 and 16 years had lower WC as adults, which was not the case when CRF only at age 13 years was considered. In contrast, when *V*O_2max_ per kg was used only CRF at age 13 was associated with lower adulthood WC, and a maintained exposure from 13 till 16 years was not statistically significant [61]. Two out of three articles that did not report on sex separately showed healthier WC in those with high CRF; one of these studies was of high quality [40]. Only two articles included pre-adolescents instead of adolescents; of those a high-quality article found that CRF was associated with lower WC [40], while in the other article there was no association [68]. Three articles measured CRF objectively and reported no association in boys and girls [60], while higher absolute *V*O_2max_ from age 13 till 16 years and *V*O_2max_ per kg at age 13 in girls was associated with lower WC [61], as was also the case in both sexes combined [33].

When waist-to-hip ratio (WHR) was the outcome of interest, one high-quality article showed no significant association between CRF and WHR for both boys and girls [55], while the high-quality article reporting on both sexes combined reported a significant inverse association [40], which was not found in a moderate-quality article [61]. Two articles included adolescents and showed no associations between CRF and WHR [55, 61]. One article used objectively measured CRF and found no association [61]. Lastly, only one high-quality article reported on waist-to-height ratio (WHtR) and showed that those with low CRF before adolescence had increased odds of high WHtR 2 years later (not shown in Table 4) [40].

### Body Composition

For boys, three out of five articles showed a significant association between higher CRF and lower body fatness [54, 56, 68], and also in one high-quality article [65]. In girls, CRF was inversely associated with body fatness in three articles [54, 56, 60], while no association was found in two others including one high-quality article [65]. Out of five articles reporting on both sexes together, one high-quality article showed a significant inverse association between CRF and fat mass and %BF [53]. The other articles showed similar significant associations [33, 37, 45, 61]. In adolescents, no association was found for boys, but in girls higher CRF was associated with lower body fatness [60], and in both sexes combined higher CRF was associated with lower body fatness [33, 61]. In articles with objectively measured CRF conflicting results for boys and girls were found [60, 65], while in both sexes combined higher CRF was associated with lower body fatness [33, 45, 61], except when *V*O_2max_ was expressed as absolute values instead of per kg of body mass [61].

### Blood Pressure

One high-quality article reported no significant association between CRF and systolic blood pressure (SBP) for boys and girls separately [55]. For boys, two out of six articles showed a significant inverse association, which in Twisk et al. was only reported for absolute *V*O_2max_ at age 13 years [61]. In girls, none of the five articles reported a significant association. In four articles that reported on both sexes combined no associations were found, except that Grontved et al. reported a significant association for one specific hemodynamic variable included in their fitness test (rate pressure product) with future SBP [33, 42, 46, 48]. All articles that separately reported on associations in boys and girls included adolescents. When findings in boys and girls were not reported separately, one article included adolescents and showed no association between CRF and SBP [33]. Three articles reported no association between objectively assessed CRF and SBP [42, 60, 61]; however, rate pressure product was found to be associated with lower SBP [42].

No high-quality articles reported on diastolic blood pressure (DBP). Two out of five articles reported a significant association in boys [35, 54]. In girls and when both sexes were combined, none of the seven articles reported a significant association between CRF and later DBP. Two articles included pre-adolescents [48, 54], of which one showed that higher CRF was associated with lower DBP in boys only [54]. Three articles showed no association of objectively measured CRF and DBP [33, 60, 61].

### Lipid Profile

One high-quality article reported no significant association between higher CRF and lower total cholesterol (TC) for boys and girls separately [55]. Similar findings were reported in two other articles [56, 60]. Furthermore, for both boys and girls combined, no significant associations between CRF and TC were found in all three articles [33, 48, 61]. All but one reported on adolescents [48], and three articles used objectively measured CRF [33, 60, 61]; none reported an association between CRF and TC.

Regarding the outcome high-density lipoprotein cholesterol (HDL-C), three articles reported separately for boys and girls and found no significant associations [56, 60, 62]. For boys and girls combined also no significant associations were found in three articles [33, 52, 61], of which one was of high quality [52]. One article reported on pre-adolescents [52], and three articles used objectively measured CRF [33, 60, 61]; none reported an association between CRF and HDL-C.

When the TC:HDL ratio was the outcome of interest, in boys three out of five articles showed a significant association between a higher CRF and lower TC:HDL ratio [54, 56, 70]. However, this was only true for 15-year-olds and not 12-year-olds [56], or when *V*O_2max_ was expressed per kg body mass and not per kg FFM [70]. In girls, two out of four articles found a significant inverse association; however, in Boreham et al. this was only for CRF measured at 12 years and not at 15 years old [56]. The two articles reporting on both sexes together showed no association between CRF and TC:HDL ratio. Four articles included adolescents, of which one showed disparate results between boys and girls at age 12 or 15 years [56], while the others reported no association [33, 60, 61]. Four articles objectively assessed CRF [33, 60, 61, 70], and only higher *V*O_2max_ per kg of body mass in boys was associated with lower TC:HDL ratio [70].

Triglycerides (TG) were the outcome of interest in one high-quality article, and no significant associations for boys and girls separately was found [52]. Similarly, no significant associations were found for boys in the three other articles, except for Latt et al. where a significant association was reported between higher *V*O_2max_ per kg body mass but not per kg of FFM and lower TG [70]. In girls, there was a significant association in one article [60], while the other article showed no association [62]. When both sexes were combined there were no significant associations in two articles [33, 46]. Three articles reported on adolescents; no associations between CRF and TG were reported in boys and both sexes combined [33, 62], while an inverse association was found in girls in one article [60]. Two articles objectively assessed CRF [33, 60]; no associations were found for boys and both sexes combined, but in girls higher CRF was associated with lower TG [60].

Lastly, CRF in pre-adolescents was not associated with low density lipoprotein cholesterol (LDL-C) in one high-quality article in the adjusted model for boys and girls separately (not shown in Table 4) [52].

### Glucose Homeostasis

CRF was not associated with glucose in boys and girls separately [62], or when they were combined [33, 59]. All three articles reported on adolescents. The two articles that objectively assessed CRF found no association for both sexes [33, 59]. For the homeostatic model of insulin resistance (HOMA-IR), all articles reported on boys and girls together. Two high-quality articles showed no association between CRF and HOMA-IR [43, 66], but low CRF was associated with increased odds of belonging to the top 75th percentile of HOMA-IR [66]. High CRF was also significantly associated with lower HOMA-IR in two articles [59, 70], although this was not true for *V*O_2max_ expressed per kg FFM instead of per kg body mass [70]. The other article did not show an association between CRF and HOMA-IR [46]. One article including adolescents reported that higher CRF was associated with lower HOMA-IR [59]. Three articles objectively measured CRF. One high-quality article showed no association [43], while two reported that higher CRF was associated with lower HOMA-IR [59, 70]; of these two studies, one reported an inverse association only when *V*O_2max_ was expressed per kg of body mass and not per kg of FFM [70]. Furthermore, pre-adolescent CRF was not significantly associated with insulin sensitivity determined by the Matsuda index in a high-quality article [43]. However, objectively measured CRF in adolescents was significantly associated with insulin and HOMA-B (beta-cell function) for both sexes (not shown in Table 4) [59].

### Metabolic Syndrome and Risks Scores

Two articles reported on the association between CRF and metabolic syndrome in boys and girls separately. One article found cut-off values of CRF predicted metabolic syndrome for boys aged 12, 13 and 14 years, and girls aged 12 and 13 years, but not for 14-year-old girls [67]. Another article only reporting on boys found no association [62]. Of the articles reporting on boys and girls combined, three showed an association between higher CRF and lower risk of metabolic syndrome [39, 50, 69], of which Schmidt et al. was of high quality [69]. Five other articles showed no association. All five articles including adolescents reported no association between CRF and the development of metabolic syndrome [33, 38, 57, 60, 62]. Objectively measured CRF was used in five articles in both sexes combined [33, 38, 39, 57, 60], of which one showed an inverse association between CRF and metabolic syndrome [33, 38, 39, 57, 60].

### Other Outcomes

Two moderate- and one high-quality article reported on other outcomes, which are not shown in Table 4. No association between objectively measured CRF and left ventricular mass in pre-adolescent boys was found, but in girls peak oxygen uptake explained 35% of the variability in left ventricular mass [44]. Sun et al. assessed high sensitivity C-reactive protein (hsCRP) and fibrinogen in pre-adolescents, showing an inverse association between CRF and fibrinogen in both sexes, and with hsCRP in girls, but not in boys [34]. Finally, a high-quality article showed that out of many arterial properties, objectively measured high CRF in adolescence was associated with reduced carotid intima media thickness (IMT) in boys, and with increased femoral artery diameter and stiffness in both sexes [58].

## Discussion

We showed that higher physical fitness, specifically CRF, during childhood and adolescence was associated with lower BMI, lower waist circumference, lower body fatness and a lower prevalence of metabolic syndrome later in life. There was no convincing evidence of an association between CRF in children and adolescents and future WHR, blood pressure, lipid profile, and glucose homeostasis. Of the articles reporting a significant association between CRF and CVD risk factors, all showed that a higher CRF was associated with lower future CVD risk factors; none of the included articles reported that higher CRF was associated with increased CVD risk factors. We therefore infer that the overall longitudinal association between CRF and CVD risk factors is probably weak to moderate. Since the most convincing evidence was found for an association between early life CRF and future adiposity, efforts to improve CRF from childhood onwards might improve the overall burden of CVD by reducing adiposity.

There are many factors affecting the variability of CRF, and a large proportion of these have a genetic origin [25]. Moderate to vigorous PA and adiposity are thought to be the largest influencers of CRF, together with sex and age [22, 71, 72]. Similarly, adiposity together with duration and intensity of PA are important factors in the development of CVD [73, 74]. Cross-sectional studies show strong correlations between CRF, PA, BMI, and CVD risk factors [17–19], meaning that these factors at time of outcome measurement might overshadow potential longitudinal associations. In particular, changes in body fatness greatly influence longitudinal associations of CRF with CVD risk factors. For instance, high adiposity at baseline resulted in lower fitness after 2 years, but reducing adiposity over the 2-year period resulted in CRF similar to controls [75]. In many of the included articles that adjusted for baseline adiposity, significant associations between CRF and future CVD risk factors were attenuated, e.g., Klakk et al. [46], Ortega et al. [51], and Grontved et al. [59]. Furthermore, adiposity at baseline significantly impacted performance in fitness testing, possibly because the additional energy required to move a larger body mass hampers individuals' ability to attain a similar level as their normal weight peers, and thus is a not necessarily a reflection of a lower level of cardiorespiratory functioning [76–78]. This could limit the ability of certain fitness (field) tests to adequately determine CRF in obese children and adolescents, and might have obscured associations between CRF and future CVD risk factors. To illustrate this, associations altered when *V*O_2max_ was expressed relative to body mass instead of relative to fat-free mass [50, 70]. However, when adiposity at follow-up was taken into account, CRF was more strongly associated with future BP in one article [35]. Despite the large biological effect of adiposity on CRF and outcome measures, not all articles adjusted for this confounder, which could have reduced the reliability of the findings.

Levels of PA and CRF are not stable and decline when children grow older [79], which could be both environmentally determined and a biological effect [80]. Associations between CRF, PA, and CVD risk factors are intricate and are probably bidirectional [81, 82]. These intricate relations could be explained in terms of those with low CRF being possibly less inclined to perform PA, resulting in reduced energy expenditure and increasing adiposity [83]. However, the opposite might also be true, i.e., adipose children might be less inclined to perform PA and therefore have decreased CRF [84]. This reverse causality could explain the varying effectiveness of childhood and adolescent PA interventions in tackling obesity [85]. This complexity and other factors influencing these relations might also explain why some authors found significant associations while others did not.

### Strengths and Limitations

A strength of this review is that stringent inclusion criteria were used. By only including children and adolescents aged 3–18 years at baseline our results were not obscured by adult participants. The minimum 2 years of follow-up ensured that a true longitudinal association was explored. There are also some limitations that warrant discussion, particularly regarding the merits of the individual studies that were available for this review. The included articles were heterogeneous with respect to methodology and measurement of CRF and outcomes, which hampered our intended meta-analysis. We attempted to convert effect estimates of individual articles so they could be pooled [86], or tried to select only articles reporting on *V*O_2max_, which proved futile. Although we were unable to weigh the effect estimates for each association, articles with smaller samples (e.g., below 100 participants) were among the articles that reported significant associations, thus suggesting that adequate sample sizes were included in these articles [33, 35, 45, 53]. The ambiguity in the reported associations might also have been due to selection bias. Many of the included articles had poor reporting on attrition, and only 11 articles were of high quality. Potentially, this could mean that individuals examined at follow-up were healthier or less healthy than the overall sample, which could have led to overestimated or underestimated associations. As with every systematic review, the quality of the data is dependent on the quality of the original articles and the way the data are reported.

In part, the heterogeneity of articles might explain the inconsistent findings. For instance, some of the included articles reported on boys and girls separately, while others corrected for sex in their models. The sex difference in CRF and prevalence of CVD risk factors would justify reporting separately for boys and girls [87]. Furthermore, differences in baseline age and duration of follow-up both could have interfered with whether significant associations were found, since CRF constantly changes during the life course [68, 88, 89]. Most studies and our review assessed CRF and outcomes at certain fixed times, and it would be interesting to specifically explore whether sustained high levels or improving CRF in children and adolescents resulted in lower future CVD risk factors.

### Recommendations

To achieve more homogeneity in future studies some recommendations are warranted. First, a uniform measure of CRF is strongly recommended. The most feasible method to express CRF seems to be as *V*O_2max_ per kg of body mass [90], since most CRF field-test results can be satisfactorily expressed as *V*O_2max_ [91, 92]. Thus, reporting CRF in both absolute values and *V*O_2max_ per kg of body mass would aid comparisons between studies. Second, the development of a core-outcome set would greatly benefit this field of research [93]. Third, we recommend reporting detailed information on the statistical methods and detailed output of the effect estimates, including confidence intervals or standard errors. Besides providing more insight into effect sizes, this would aid future systematic reviews in calculating standardized effect sizes to be pooled in meta-analyses [86]. Furthermore, specifics and outcomes of unadjusted and adjusted models should be reported. Fourth, to clarify attrition rates authors should adhere to reporting guidelines such as the STROBE guideline [31]. Last, since CRF and most of the outcomes of interest are age- and sex-dependent, we would recommend use of age adjusted z-scores when possible [94], and reporting on boys and girls separately, even if there were no differences in the associations found [95].

Future systematic reviews on this topic should aim to gather individual patient fitness data at baseline and outcome data at follow-up [96]. This would give the authors the ability to infer stronger conclusions and if possible adjust for potential confounders, such as adiposity [97, 98]. Unfortunately, we did not account for this during the planning of this systematic review.

## Conclusion

We showed that higher CRF in childhood and adolescence is associated with lower BMI, body fatness, and metabolic syndrome incidence at least 2 years later. For WHR, blood pressure, lipid metabolism and glucose homeostasis the evidence is unconvincing. These findings could be hampered by confounders that were not uniformly accounted for, such as adiposity at baseline and/or follow-up. High CRF in children and adolescents was not linked to increased CVD risk factors in any of the articles. Addressing CRF in children and adolescents could reduce future adiposity and thus be an important factor in improving health. Recommendations for future research include standardizing the measurement of CRF, reporting standardized outcome assessments, and performing individual patient data meta-analyses.

## Electronic supplementary material

Below is the link to the electronic supplementary material.

Supplementary material 1 (DOCX 33 kb)
