# Supplementary material for: Cardiorespiratory Fitness in Childhood and Adolescence Affects Future Cardiovascular Risk Factors: A Systematic Review of Longitudinal Studies
Source: Sports Med. 2018 Aug 24;48(11):2577–605. doi: 10.1007/s40279-018-0974-5 (PMC6182463; doi:10.1007/s40279-018-0974-5)
Supplement: Supplementary file 1 — Supplementary material 1 (DOCX 33 kb) [file 40279_2018_974_MOESM1_ESM.docx]

# Electronic Supplementary Material Appendix S1

MEDLINE(R) Epub Ahead of Print, In-Process & Other Non-Indexed Citations, Ovid MEDLINE(R) Daily and Ovid MEDLINE(R) 1946 to Present (Ovid, 23 October 2017):

1. exp child/ or exp adolescent/ or minors/ or exp schools/ or exp puberty/
2. (youngster or pubert* or pubescent or prepubescent or school or schools or schoolkid* or schoolchild* or highschool* or kid or kids or underage* or youth? or boy or boys or girl? or sibbling* or preschool* or child or children or schoolchild* or adolescents or adolescence or juvenile or minors or teen or teens or teenager* or p?ediatric?).ab,kf,ti.
3. (child or p?ediatric? or adolescents or adolescence or juvenile).jn.
4. or/1-3 [Children aged 3-18 y]
5. exp athletic performance/
6. ((cardio-respiratory or cardiorespiratory or aerobic or cardiopulmonary or cardiovascular) adj2 (capacit* or endurance or fitness)).ab,kf,ti.
7. (physical fitness or Exercise capacit* or Physical work capacit* or Exercise tolerance or Exercise test? or Maximal exercise? or Exercise response? or Aerobic power).ab,kf,ti.
8. or/5-7 [fitness]
9. exercise tests/ or (exercise/ and longitudinal studies/)
10. (Field test? or Shuttle run test? or Shuttle walk test? or Cooper test? or "run/walk" or walking test? or Multi stage running test? or ALPHA field test? or EUROFIT or PREFIT battery or pre-fit battery or FITNESSGRAM or PCHF or presidents challenge or president's challenge or PCPF or AAUTB or Athletic union or YMCAYFT or YMCA young fitness test? or NYPFP or National youth physical program or HRFT or health related fitness test? or Physical best or IPFT or International physical fitness test? or "CAHPER-FPT II" or fitness perfmance test? or CPAFLA or canadian physical activity or NFTP-PRC or national fitness test? program or NZFT or new Zealand fitness test? or AFEA or Australian fitness education award).ab,kf,ti.
11. or/9-10 [tests]
12. (metabolic equivalent units or oxygen consumption or VO2 max or VO2 peak or VO2 plateau or Relative oxygen uptake or Respiratory exchange rate or Lactate threshold or Ventilatory threshold or Fick equation).ab,kf,ti. [parameters]
13. exp longitudinal studies/
14. (measure* adj5 (year? or month*)).ab,kf,ti.
15. (longitudinal or follow-up or prospective or predictive or course).ab,kf,ti.
16. or/13-15 [longitudinal studies]
17. 4 and (8 or 11 or 12) and 16
18. (young hearts study or young hearts project or young hearts cohort).ab,kf,ti.
19. 17 or 18
20. (anthropometr* or obese or obesity or overweight or weight or fatness or metabolic syndrome or syndrome X or body composition or bodycomposition or adiposit* or body mass or body mass index or BMI or fatmass or percentage fat or body fat or waist circumference or waist hip ratio or hip circumference or skinfold*).ab,kw,ti. [anthropometry]
21. (cardiovascular or cardiometabolic or noncommunicable or heart or heartdisease or vascular disease or myocardial ische?mia or coronary atherosclerosis or cardiovascular risk or Framingham risk or CVD or risk factors or risk score or blood pressure or hypertension or systolic or diastolic).ab,kw,ti. [heart disease]
22. (arterial stiffness or aortic stiffness or arteries or aorta or pulse wave velocity or central pulse wave or augmentation index or IMT or intima media thickness or microcirculation or endothel* or endothelial dysfunction or flow mediated dilatation or endothelial mediated vasodilatation or laser Doppler flowmetry or laser Doppler fluximetry or macro circulation or macrocirculation or circulation).ab,kw,ti. [vascular]
23. (metabolic or insulin or glycemic or glucose or lipid* or lipoprotein* or cholesterol or HDL or high-density lipoprotein or cholesterol or LDL or low-density lipoprotein or triglycerides or TG or adipokines or blood lipids or dyslipid?emia or diabetes or inflammat*).ab,kw,ti. [metabolic]
24. 19 and (20 or 21 or 22 or 23)
25. animals/ not humans/
26. 24 not 25
27. ..dedup 26

Embase Classic+Embase (Ovid interface, 23 October 2017):

1. exp *child/ or *adolescent/ or *school child/ or *school/ or *college/ or *high school/ or *middle school/ or *nursery school/ or exp *adolescence/ or *child health/ or *childhood/ or *pediatrics/ or *juvenile/ or *exp adolescent health/ or *"minor (person"/ or *adopted child/ or *school health service/
2. (youngster or pubert* or pubescent or prepubescent or school or schools or schoolkid* or schoolchild* or highschool* or kid or kids or underage* or youth? or boy or boys or girl? or sibbling* or preschool* or child or children or schoolchild* or adolescents or adolescence or juvenile or minors or teen or teens or teenager* or p?ediatric?).ab,kw,ti.
3. (child or p?ediatric? or adolescents or adolescence or juvenile).jn.
4. or/1-3 [Children aged 3-18 y]
5. *athletic performance/ or *cardiorespiratory fitness/ or *cardiopulmonary exercise test/ or *cardiopulmonary function/ or *aerobic exercise/
6. ((cardio-respiratory or cardiorespiratory or aerobic or cardiopulmonary or cardiovascular) adj2 (capacit* or endurance or fitness)).ab,kw,ti.
7. (physical fitness or Exercise capacit* or Physical work capacit* or Exercise tolerance or Exercise test? or Maximal exercise? or Exercise response? or Aerobic power).ab,kw,ti.
8. or/5-7 [fitness]
9. *exercise test/ or (exp *exercise/ AND *longitudinal study/)
10. (Field test? or Shuttle run test? or Shuttle walk test? or Cooper test? or "run/walk" or walking test? or Multi stage running test? or ALPHA field test? or EUROFIT or PREFIT battery or pre-fit battery or FITNESSGRAM or PCHF or presidents challenge or president's challenge or PCPF or AAUTB or Athletic union or YMCAYFT or YMCA young fitness test? or NYPFP or National youth physical program or HRFT or health related fitness test? or Physical best or IPFT or International physical fitness test? or "CAHPER-FPT II" or fitness performance test? or CPAFLA or canadian physical activity or NFTP-PRC or national fitness test? program or NZFT or new Zealand fitness test? or AFEA or Australian fitness education award or ergometry or ergometer or treadmill test?).ab,kw,ti.
11. or/9-10 [tests]
12. (metabolic equivalent units or oxygen consumption or VO2 max or VO2 peak or VO2 plateau or Relative oxygen uptake or Respiratory exchange rate or Lactate threshold or Ventilatory threshold or Fick equation).ab,kw,ti. [parameters]
13. (measure* adj5 (year? or month*)).ab,kw,ti.
14. (longitudinal or follow-up or prospect* or predict* or course).ab,kw,ti.
15. or/13-14 [longitudinal studies]
16. 4 and (8 or 11 or 12) and 15
17. (young hearts study or young hearts project or young hearts cohort).ab,kw,ti.
18. 16 or 17
19. (animal/ or animal experiment/ or animal model/ or nonhuman/ or rat/ or mouse/ or (rat or rats or mouse or mice).ti.) not human/
20. 18 not 19
21. limit 20 to (book or book series or conference abstract or conference proceeding or "conference review")
22. 20 not 21
23. ..dedup 22

SPORTDiscus (Ebscohost, 23 October 2017):

S16 S12 OR S15

S15 S4 AND S13 AND S14

S14 S5 OR S6 OR S7 OR S8 OR S9

S13 S10 OR S11

S12 SU young hearts study or young hearts project or young hearts cohort OR AB young hearts study or young hearts project or young hearts cohort OR TI young hearts study or young hearts project or young hearts cohort

S11 SU longitudinal or follow-up or prospective or predictive or course OR AB longitudinal or follow-up or prospective or predictive or course OR TI longitudinal or follow-up or prospective or predictive or course

S10 SU measure* N5 (year? or month*) OR AB measure* N5 (year? or month*) OR TI measure* N5 (year? or month*)

S9 SU metabolic equivalent units or oxygen consumption or VO2 max or VO2 peak or VO2 plateau or Relative oxygen uptake or Respiratory exchange rate or Lactate threshold or Ventilatory threshold or Fick equation OR AB metabolic equivalent units or oxygen consumption or VO2 max or VO2 peak or VO2 plateau or Relative oxygen uptake or Respiratory exchange rate or Lactate threshold or Ventilatory threshold or Fick equation OR TI metabolic equivalent units or oxygen consumption or VO2 max or VO2 peak or VO2 plateau or Relative oxygen uptake or Respiratory exchange rate or Lactate threshold or Ventilatory threshold or Fick equation

S8 SU Field test? or Shuttle run test? or Shuttle walk test? or Cooper test? or "run/walk" or walking test? or Multi stage running test? or ALPHA field test? or EUROFIT or PREFIT battery or pre-fit battery or FITNESSGRAM or PCHF or presidents challenge or president's challenge or PCPF or AAUTB or Athletic union or YMCAYFT or YMCA young fitness test? or NYPFP or National youth physical program or HRFT or health related fitness test? or Physical best or IPFT or International physical fitness test? or "CAHPER-FPT II" or fitness perfmance test? or CPAFLA or canadian physical activity or NFTP-PRC or national fitness test? program or NZFT or new Zealand fitness test? or AFEA or Australian fitness education award OR AB Field test? or Shuttle run test? or Shuttle walk test? or Cooper test? or "run/walk" or walking test? or Multi stage running test? or ALPHA field test? or EUROFIT or PREFIT battery or pre-fit battery or FITNESSGRAM or PCHF or presidents challenge or president's challenge or PCPF or AAUTB or Athletic union or YMCAYFT or YMCA young fitness test? or NYPFP or National youth physical program or HRFT or health related fitness test? or Physical best or IPFT or International physical fitness test? or "CAHPER-FPT II" or fitness perfmance test? or CPAFLA or canadian physical activity or NFTP-PRC or national fitness test? program or NZFT or new Zealand fitness test? or AFEA or Australian fitness education award OR TI Field test? or Shuttle run test? or Shuttle walk test? or Cooper test? or "run/walk" or walking test? or Multi stage running test? or ALPHA field test? or EUROFIT or PREFIT battery or pre-fit battery or FITNESSGRAM or PCHF or presidents challenge or president's challenge or PCPF or AAUTB or Athletic union or YMCAYFT or YMCA young fitness test? or NYPFP or National youth physical program or HRFT or health related fitness test? or Physical best or IPFT or International physical fitness test? or "CAHPER-FPT II" or fitness perfmance test? or CPAFLA or canadian physical activity or NFTP-PRC or national fitness test? program or NZFT or new Zealand fitness test? or AFEA or Australian fitness education award

S7 DE "EXERCISE tests" OR DE "STRESS echocardiography" OR DE "TREADMILL exercise tests"

S6 SU physical fitness or Exercise capacit* or Physical work capacit* or Exercise tolerance or Exercise test? or Maximal exercise? or Exercise response? or Aerobic power or AB physical fitness or Exercise capacit* or Physical work capacit* or Exercise tolerance or Exercise test? or Maximal exercise? or Exercise response? or Aerobic power OR TI physical fitness or Exercise capacit* or Physical work capacit* or Exercise tolerance or Exercise test? or Maximal exercise? or Exercise response? or Aerobic power

S5 SU (cardio-respiratory or cardiorespiratory or aerobic or cardiopulmonary or cardiovascular) N2 (capacit* or endurance or fitness) OR AB (cardio-respiratory or cardiorespiratory or aerobic or cardiopulmonary or cardiovascular) N2 (capacit* or endurance or fitness) or TI (cardio-respiratory or cardiorespiratory or aerobic or cardiopulmonary or cardiovascular) N2 (capacit* or endurance or fitness)

S4 S1 OR S2 OR S3

S3 JN child or p?ediatric? or adolescents or adolescence or juvenile

S2 SU youngster or pubert* or pubescent or prepubescent or school or schools or schoolkid* or schoolchild* or highschool* or kid or kids or underage* or youth? or boy or boys or girl? or sibbling* or preschool* or child or children or schoolchild* or adolescents or adolescence or juvenile or minors or teen or teens or teenager* or p?ediatric? or AB youngster or pubert* or pubescent or prepubescent or school or schools or schoolkid* or schoolchild* or highschool* or kid or kids or underage* or youth? or boy or boys or girl? or sibbling* or preschool* or child or children or schoolchild* or adolescents or adolescence or juvenile or minors or teen or teens or teenager* or p?ediatric? or TI youngster or pubert* or pubescent or prepubescent or school or schools or schoolkid* or schoolchild* or highschool* or kid or kids or underage* or youth? or boy or boys or girl? or sibbling* or preschool* or child or children or schoolchild* or adolescents or adolescence or juvenile or minors or teen or teens or teenager* or p?ediatric?

S1 DE "CHILDREN" OR DE "AIDS (Disease) & children" OR DE "CHILD acrobats" OR DE "CHILD circus performers" OR DE "CHILD dancers" OR DE "CHILD development" OR DE "DANCE for children" OR DE "DEAFBLIND children" OR DE "OUTDOOR recreation for children" OR DE "OVERWEIGHT children" OR DE "SCHOOL children" OR DE "SELF-defense for children" OR DE "VIDEO games & children" OR DE "SCHOOL children" OR DE "CHILDREN -- Health" OR DE "PEDIATRICS" OR DE "TEENAGERS" OR DE "YOUTH"
